# Extracorporeal carbon dioxide removal for patients with acute respiratory failure secondary to the acute respiratory distress syndrome: a systematic review

**DOI:** 10.1186/cc13875

**Published:** 2014-05-15

**Authors:** Marianne Fitzgerald, Jonathan Millar, Bronagh Blackwood, Andrew Davies, Stephen J Brett, Daniel F McAuley, James J McNamee

**Affiliations:** 1 Centre for Infection and Immunity, School of Medicine, Dentistry and Biomedical Science, Queen’s University Belfast, 97 Lisburn Road, Belfast BT9 7AE, UK

## Abstract

Acute respiratory distress syndrome (ARDS) continues to have significant mortality and morbidity. The only intervention proven to reduce mortality is the use of lung-protective mechanical ventilation strategies, although such a strategy may lead to problematic hypercapnia. Extracorporeal carbon dioxide removal (ECCO_2_R) devices allow uncoupling of ventilation from oxygenation, thereby removing carbon dioxide and facilitating lower tidal volume ventilation. We performed a systematic review to assess efficacy, complication rates, and utility of ECCO_2_R devices. We included randomised controlled trials (RCTs), case–control studies and case series with 10 or more patients. We searched MEDLINE, Embase, LILACS (Literatura Latino Americana em Ciências da Saúde), and ISI Web of Science, in addition to grey literature and clinical trials registries. Data were independently extracted by two reviewers against predefined criteria and agreement was reached by consensus. Outcomes of interest included mortality, intensive care and hospital lengths of stay, respiratory parameters and complications. The review included 14 studies with 495 patients (two RCTs and 12 observational studies). Arteriovenous ECCO_2_R was used in seven studies, and venovenous ECCO_2_R in seven studies. Available evidence suggests no mortality benefit to ECCO_2_R, although *post hoc* analysis of data from the most recent RCT showed an improvement in ventilator-free days in more severe ARDS. Organ failure-free days or ICU stay have not been shown to decrease with ECCO_2_R. Carbon dioxide removal was widely demonstrated as feasible, facilitating the use of lower tidal volume ventilation. Complication rates varied greatly across the included studies, representing technological advances. There was a general paucity of high-quality data and significant variation in both practice and technology used among studies, which confounded analysis. ECCO_2_R is a rapidly evolving technology and is an efficacious treatment to enable protective lung ventilation. Evidence for a positive effect on mortality and other important clinical outcomes is lacking. Rapid technological advances have led to major changes in these devices and together with variation in study design have limited applicability of analysis. Further well-designed adequately powered RCTs are needed.

## Introduction

Acute respiratory distress syndrome (ARDS) is associated with significant mortality and morbidity [1]. Tidal recruitment, alveolar derecruitment and high inspiratory volumes during mechanical ventilation add further insult to already injured and failing lungs [2]. Few interventions have been proven to reduce mortality, with the notable exception of low tidal volume ventilation [3]. In practice, lung-protective strategies involving low tidal volume ventilation can prove difficult to implement, often due to concerns of hypercapnia or its potential adverse physiological consequences [4].

Extracorporeal carbon dioxide removal (ECCO_2_R) offers a potentially attractive solution to this problem because carbon dioxide can be ‘dialysed’ out of the blood, while the lungs are ventilated in a maximally protective manner [5]. Techniques to achieve this have existed since the late 1970s [6,7], but widespread uptake has been limited due to the paucity of trial data, the demanding technical requirements of the technique and concerns regarding complications [8]. More recently, modern developments in ECCO_2_R technology have stimulated renewed interest [9], particularly because of the potential for safe use in nonspecialist centres. To define current understanding of ECCO_2_R in patients with acute respiratory failure and inform future randomised controlled trials (RCTs), we performed a systematic review to assess efficacy and complication rates of ECCO_2_R.

## Review

### Design

The systematic review protocol was published in the PROSPERO database [10] and complies with Preferred Reporting Items for Systematic reviews and Meta-Analysis guidelines [11].

We searched the MEDLINE, Embase, LILACS (Literatura Latino Americana em Ciências da Saúde) and ISI Web of Science databases (1976 to January 2014) using a strategy developed by a trained medical librarian, combining medical subject headings and keywords such as interventional lung assist, extracorporeal and ARDS (see Additional file 1 for the full MEDLINE search strategy). Citations were screened by title/abstract level, and if they were potentially relevant the full text was retrieved and reviewed by MF and JM. We also reviewed reference lists of identified studies and relevant review papers.

The search of grey literature included Opengray, NHS Evidence, National Institute of Clinical Evidence and the Scottish Intercollegiate Guidelines Network. To identify planned or current studies we examined major clinical trial registries [12-14]. Novalung GmbH (Talheim, Germany), who manufacture the interventional lung assist and iLA Activve®, were consulted to identify any current studies. The search strategy included no language restriction.

Inclusion criteria were: type of study – RCT or observational (for example, case–control or case series) including 10 or more patients; type of participants – adult patients (>18 years) with ARDS (or acute respiratory failure in studies occurring prior to the American–European Consensus Conference Committee definition of ARDS in 1994); type of interventions – arteriovenous or venovenous ECCO_2_R device; and type of outcomes – hospital or ICU mortality, hospital or ICU length of stay, ventilator-free days (VFDs), organ failure-free days, quantified carbon dioxide removal, and reported complications.

### Study selection

Two authors (MF and JM) independently reviewed the retrieved abstracts and assessed eligibility. Full-text articles were retrieved and assessed to confirm eligibility. Disagreement was resolved by consensus with a third author (DFM).

### Data extraction

Data from included studies were independently extracted by MF and JM, using a pre-piloted data extraction form (Additional file 2). Disagreements were resolved by a third author (BB). We extracted the following data: study design, study and participant characteristics, study intervention and setting, relevant outcome data, and complications.

### Assessment of methodological quality

Two authors (MF and JM) independently assessed methodological quality. Observational studies were assessed using the Critical Appraisal Skills Programme tools and, for case series, a specific checklist [15,16]. RCTs were assessed using the risk of bias domain-based evaluation as described in the Cochrane Handbook for Systematic Reviews of Interventions [17].

### Search results

**Figure 1:**
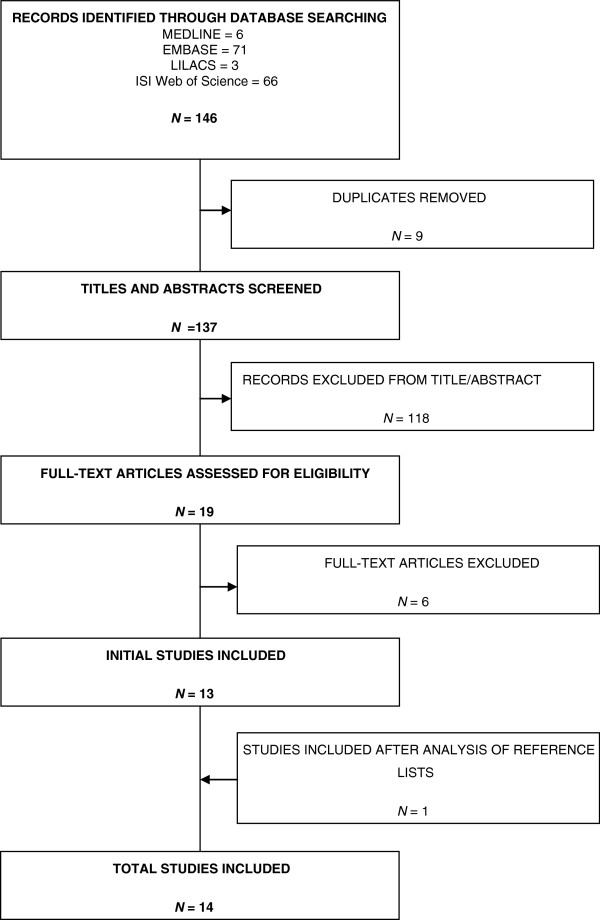
**Literature search.** LILACS, Literatura Latino Americana em Ciências da Saúde.

The search identified 147 citations from database searches and reference lists (Figure 1). Following removal of duplicates (*n* = 9), removal of those not meeting screening eligibility criteria (*n* = 118) and full-text exclusions (*n* = 6; see Additional file 3), 14 studies were included in this review (two RCTs and 12 observational studies) [18-31].

A search of the grey literature revealed two abstracts presented at an international conference [32,33]. The first was for a retrospective analysis of 325 patients with ARDS and acute kidney injury treated with continuous renal replacement therapy with or without ECCO_2_R [32]. The second abstract described a study of 10 patients using percutaneous extracorporeal lung assist combined with continuous renal replacement therapy [33]. Limited information was provided in both cases, and so neither abstract was included in our analysis.

One health technology assessment of an arteriovenous ECCO_2_R device was identified [34].

### Characteristics of included studies

In total, 14 studies were included: two RCTs (Table 1) [18,19], and six prospective observational studies [20-25] and six retrospective studies [26-31] (Table 2). The earliest study dated from 1986 and the most recent from 2013. Among included studies, nine were from Germany, two were from France, two were from Italy, and one was from the United States. All of the studies were published in peer-reviewed journals. Only one study was multicentre in design [19]. The population studied was defined as ARDS in 13 studies and acute respiratory failure in one study [24].

**Table 1:** Description of included randomised controlled trials

**Study**	**Year**	***N***	**Intervention**	**Control**	**Mean age**^**a**^	**Male/ female**^**a**^	**Primary outcome measure**	**Secondary outcome measures**	**VFD**^**b**^			**Mortality**^**c**^		
									**I**	**C**	***P *** **value**	**I**	**C**	***P *** **value**
Bein and colleagues [19]	2013	79	iLA	ARDSnet ventilation	49.8 ± 12 (48.7 ± 17)	38/2 (30/9)	Ventilator-free days (28 and 60 days)	Respiratory parameters, haemodynamics, inflammatory response, transfusion requirements, analgesic/sedative requirements, catecholamine requirements, frequency and duration of RRT, organ failure-free days, frequency and duration of adjunctive therapies, complications, ICU and hospital LOS, in-hospital mortality	10 ± 8 (33.2 ± 20)	9.3 ± 9 (29.2 ± 21)	0.779 (0.469)	7/40 (17.5%)	6/39 (15.4%)	0.000
Morris and colleagues [18]	1994	40	PCIRV then LFPPV + VV ECCO_2_R	Standardised CPPV	33 ± 3.1 (35 ± 2.3)	8/13 (9/10)	30-day mortality	Respiratory parameters, transfusion requirements, complications, ICU and hospital LOS, economic analysis	NR			14/21 (66.6%)	11/19 (57.9%)	0.56

C, control group; CPPV, conventional positive pressure ventilation; I, intervention group; iLA, interventional lung assist; LFPPV, low-frequency positive-pressure ventilation; LOS, length of stay; NR, not recorded; PCIRV, pressure controlled inverse ratio ventilation; RRT, renal replacement therapy; VV ECCO_2_R, venovenous extracorporeal carbon dioxide removal. ^a^Intervention (control). ^b^Ventilator-free days at 28 days (60 days). ^c^In-hospital mortality for Bein and colleagues [19], and 30-day mortality for Morris and colleagues [18].

**Table 2:** Description of included nonrandomised studies

**Study**	**Design**	**Year**	***N***	**Intervention**	**Mean age (years)**	**Male/ female**	**Outcomes measured**	**Mortality (%)**	**Grade**
Forster and colleagues [25]	Prospective case series	2013	10	VV (with CRRT)	60	7/3	Respiratory and haemodynamic parameters, complications, mortality	40	3
Nierhaus and colleagues [29]	Retrospective case series	2011	13	AV	52	8/5	Respiratory parameters, ICU LOS, complications, mortality	54	3
Weber-Carstens and colleagues [26]	Retrospective case series	2009	10	AV	54	6/4	Respiratory parameters, sedation scores, ICU LOS, mortality	60	3
Zimmermann and colleagues [20]	Prospective case series	2009	51	AV	52	43/8	Respiratory parameters, haemodynamics, complications, mortality	49	3
Terragni and colleagues [21]	Prospective cross-sectional study	2009	32	VV	66	22/10	Respiratory parameters, lung morphology, inflammatory response, complications	NR	2+
Muellenbach and colleagues [27]	Retrospective case series	2008	22	AV	38	20/2	Respiratory parameters, haemodynamics, complications, ventilator-free days, ICU LOS, mortality	27	3
Bein and colleagues [28]	Retrospective case series	2006	90	AV	44	69/21	Respiratory parameters, haemodynamics, complications, mortality	59	3
Liebold and colleagues [30]	Retrospective case series	2002	70	AV	41	55/15	Respiratory parameters, complications, mortality	64	3
Guinard and colleagues [22]	Prospective cross-sectional study	1997	10	VV	NR	NR	Respiratory parameters, complications, mortality	75	2+
Brunet and colleagues [23]	Prospective case series	1994	11	VV	27	4/7	Respiratory parameters, mortality	27	3
Bindslev and colleagues [31]	Retrospective case series	1991	14	VV	31	11/3	Respiratory parameters, complications, mortality	57	3
Gattinoni and colleagues [24]	Prospective case series	1986	43	VV	26	18/25	Respiratory parameters, haemodynamics, complications, mortality	51	3
Total			376			263/103(+10 NR)			

AV, arteriovenous; CRRT, continuous renal replacement therapy; LOS, length of stay; NR, not recorded; VV, venovenous.

The configuration of ECCO_2_R differed across studies, with seven using arteriovenous circuits and seven using venovenous circuits. Only one study conducted after 2000 examined the use of a venovenous device [21]. In total this systematic review included analysis of 335 patient cases of arteriovenous ECCO_2_R and 160 cases of venovenous ECCO_2_R.

### Risk of bias in included studies

Given the nature of the intervention, neither RCT was blinded to allocation. Both RCTs reported an intention-to-treat analysis. Both trials scored a low risk of bias on all domains, except in Morris and colleagues’ paper where the sequence generation was unclear [18] (Table 3). Both RCT studies halted recruitment earlier than planned, as in both cases interim analysis concluded that the difference between new and control therapies was too small for a significant survival improvement to be demonstrated.

**Table 3:** Risk of bias and methodological quality assessment of included randomised controlled trials

**Study**	**Sequence generation**	**Allocation concealment**	**Selective outcome reporting**	**Other bias**	**Summary**
Bein and colleagues [19]	Low	Low	Low	Low^a^	Low
Morris and colleagues [18]	Unclear	Low	Low	Low^b^	Low

^a^Trial stopped early due to futility. ^b^Trial stopped early after an interim analysis showed treatment effect too small to be demonstrated by proposed sample size.

The Xtravent study by Bein and colleagues presented an overall low risk of bias [19] (Table 3). Although the initial sample size calculation conducted by the Xtravent trialists suggested that 120 patients (53 patients per group for a power of 0.8 and an alpha of 0.05) would need to be recruited to identify a significant increase in 28-day VFDs (the primary outcome measure), following a planned interim analysis after enrolment of 56 patients the study period was limited to 3 years because a significant difference was not likely to be shown in the planned cohort. At conclusion, the study had enrolled 79 patients.

The study by Morris and colleagues was judged to present a low risk of bias [18] (Table 3). The study aimed to recruit 60 patients, but was halted after 40 participants were enrolled. This occurred after a pre-defined interim analysis concluded that the survival difference between the intervention and control therapies was too small for a significant difference to be demonstrated with 60 randomised patients. Analysis after 40 patients found a small difference in survival between the control group and the ECCO_2_R group (mean survival, 30% in ECCO_2_R patients versus 39% in controls), and it was projected that 400 patients would be needed to demonstrate statistical significance.

### Effect of interventions

Given the variation in study designs a meta-analysis was considered to be inappropriate and data were descriptively synthesised.

### Primary outcome

Mortality was the primary outcome. Thirteen studies presented data on mortality [18-20,22-31]. Neither RCT demonstrated a statistically significant difference in hospital mortality for those undergoing ECCO_2_R. Interestingly, the latest RCT reported a relatively low mortality (control group, 15%; ECCO_2_R group, 18%), which is in keeping with mortality figures from other recent ARDS studies [35], suggesting that overall survival from ARDS is improving. The observational studies reported mortality rates ranging from 27 to 75% (mean 55.5%, standard deviation 47.2 to 60.3).

### Secondary outcomes

#### Ventilator-free days

Two studies reported VFDs [18,27]. The primary outcome measure in the Xtravent study was VFDs to 28 and 60 days [19]. Subgroup analysis of those patients with partial pressure of arterial oxygen (PaO_2_)/fraction of inspired oxygen (FiO_2_) <150 demonstrated a significant increase in VFDs in those receiving ECCO_2_R at both 28 and 60 days (mean ± standard deviation 11.3 ± 7.5 versus 5 ± 6.3 days, *P* = 0.033 and 40.9 ± 12.8 versus 28.2 ± 16.4 days, *P* = 0.033, respectively). This increase in VFDs was not seen in patients with higher PaO_2_/FiO_2_ ratios.

#### Duration of ICU stay and organ failure-free days

Five studies (two RCTs and three retrospective studies) reported ICU length of stay [18,19,26,27,29]. Neither RCT demonstrated a significant reduction in ICU length of stay. Both RCTs also reported hospital length of stay, but did not demonstrate a difference between groups.

Only the Xtravent study reported on organ failure-free days, and demonstrated no difference between groups [19].

### Respiratory parameters including carbon dioxide removal

Significant variation existed in the respiratory parameters measured among studies (Table 4). Several studies reported comparative analysis of respiratory parameters between survivors and nonsurvivors of ECCO_2_R [24,27,28], whilst other studies reported measurements at varying time points, from 2 hours after initiation of ECCO_2_R to its discontinuation.

**Table 4:** Principal respiratory outcomes in nonrandomised studies

**Study**	**Time**	**PaCO**_**2 **_**change (mmHg)**	**Change in PaO**_**2**_**/FiO**_**2 **_**(mmHg)**	**Pplat change (mmHg)**	**V**_**t **_**reduction**	**pH change**
Forster and colleagues [25]	4 hours	68.00 ± 8.28 to 49.6 ± 6.18	NR	19.8 ± 2.0 to 19.0 ± 2.4	8.41 ± 0.30 to 8.34 ± 1.04	7.18 ± 0.08 to 7.30 ± 0.07
Nierhaus and colleagues [29]	Day 1	80.0 ± 23.0 to 54.0 ± 19.0	100.0 ± 28.9 to 120.7 ± 51.2	34.0 ± 3.0^b^ to 28.3 ± 4.0	292.5 ± 94.0^a^ to 183.0 ± 67.0	7.18 ± 0.22 to 7.37 ± 0.09
Weber-Carstens and colleagues [26]	NR	120 (81.9 to 152.5) to 60.3 (52.5 to 69)	121.5 (79.3 to 178.3) to 87 (73 to 139.3)	39 (34.8 to 43.3) to 31.5 (28.8 to 33.5)	5.2 (4.4 to 5.9) to 3.5 (3.1 to 4.3)^b^	7.06 (6.9 to 7.3) to 7.38 (7.18 to 7.48)
Zimmermann and colleagues [20]	24 hours	73 (61 to 86) to 41 (34 to 48)	75 (62 to 130) to 110 (86 to 160)	35 (31 to 38) to 30 (26 to 34)	6.6 (5.3 to 7.2) to 4.4 (3.4 to 5.4)^b^	7.23 (7.16 to 7.30) to 7.44 (7.37 to 7.45)
Terragni and colleagues [21]	NR	47.2 ± 8.6 to 73.6 ± 11.1	NR	NR	NR	7.20 ± 0.02 to 7.38 ± 0.04
Muellenbach and colleagues [27]	12 hours	65.26 (54 to 72) to 39.8 (36 to 42)	61.6 (47.3 to 85.6) to 135.8 (87.8 to 153)	NR	450 (400 to 542.5) to 200 (145 to 250)^a^	7.25 (7.22 to 7.29) to 7.4 (7.37 to 7.42)
Bein and colleagues [28]	24 hours	60 (48 to 80) to 34 (30 to 39)	58 (47 to 78) to 107 (74 to 142)	NR	430 (360 to 540) to 380 (320 to 470)^a^	7.27 (7.18 to 7.36) to 7.45 (7.41 to 7.50)
Liebold and colleagues [30]	24 hours	59 ± 17 to 32 ± 8	50 to 110	NR	NR	NR
Guinard and colleagues [22]	NR	NR	NR	NR	NR	NR
Brunet and colleagues [23]	NR	66 ± 25 to 43 ± 6	79 ± 21 to 207 ± 108	48 ± 10 to 37 ± 5	622 ± 131 to 270 ± 60^a^	NR
Bindslev and colleagues [31]	NR	NR	NR	NR	NR	NR
Gattinoni and colleagues [24]	NR	NR	NR	NR	NR	NR

Data presented as mean ± standard deviation or median (range). NR, not recorded; FiO_2_, fraction of inspired oxygen; PaCO_2_, partial pressure of arterial carbon dioxide; PaO_2_, partial pressure of arterial oxygen; Pinsp, inspiratory pressure; Pplat, plateau pressure; V_t_, tidal volume. ^a^Units are millilitres. ^b^Units are millilitres/kilogram.

The Xtravent study demonstrated a significant reduction in tidal volume, minute ventilation and ΔP (plateau pressure – positive end-expiratory pressure) in the ECCO_2_R group, which was sustained across several days of therapy [19]. Morris and colleagues also reported a sustained reduction in tidal volume in those undergoing ECCO_2_R, with an initial improvement in peak inspiratory pressures [18]. In both RCTs, tidal volumes approaching 3 ml/kg predicted body weight were achieved at least in the initial period after initiation of ECCO_2_R.

Nonrandomised studies reported outcomes at varying times after commencing ECCO_2_R ranging from 2 hours [28] to decannulation [29]. In the early period (to day 1), all showed reductions in tidal volume, peak inspiratory pressure, arterial partial pressure of carbon dioxide and increase in arterial pH. The level of positive end-expiratory pressure administered to patients remained largely unchanged in the majority of studies. The PaO_2_/FiO_2_ ratio increased in all but three studies [19,24,25]. Carbon dioxide removal was quantitatively possible using strategies of arteriovenous carbon dioxide removal [19,21,26,29] (Table 4).

### Complications

All but one study [22] reported on complications encountered with ECCO_2_R therapy (Table 5). Complication rates ranged from 0 to 25%. Five studies reported rates in excess of 20% [21,27-30]. Amongst studies examining arteriovenous devices, the most common complication was lower limb ischaemia secondary to arterial cannulation. In the majority of studies this was a transient complication, but five cases of compartment syndrome and one case of lower limb amputation were reported. In studies where venovenous ECCO_2_R was used, clotting within the circuit is the main complication, with catheter and membrane malfunction also reported. Studies conducted before 2000 (all using venovenous ECCO_2_R; Table 5) report higher rates of diffuse bleeding or at sites other than that where cannula insertion has occurred.

**Table 5:** Complications of extracorporeal carbon dioxide removal

**Study**	**Year**	**Modality**	**Complication rate (%)**	**Description**
Forster and colleagues [25]	2013	VV (with CRRT)	Nil	
Bein and colleagues [19]	2013	AV	7.5	1× lower limb ischaemia, 2× aneurysm
Nierhaus and colleagues [29]	2011	AV	21.4	2× catheter displacement, 1× bleeding at insertion site
Weber-Carstens and colleagues [26]	2009	AV	6.3	1× transient occlusion of femoral artery during cannulation
Zimmermann and colleagues [20]	2009	AV	11.8	3× lower limb ischaemia, 1× cannula thrombosis, 1× bleeding during cannulation, 1× compartment syndrome
Terragni and colleagues [21]	2009	VV	25	1× pump malfunction, 3× membrane/haemofilter clotting, 1× catheter displacement, 3× cannula problems
Muellenbach and colleagues [27]	2008	AV	23	2× lower limb ischaemia, 1× femoral artery pseudoaneurysm, 1× lower limb amputation, 1× catheter displacement
Bein and colleagues [28]	2006	AV	24.4	9× lower limb ischaemia, 4× cannula thrombosis, 4× compartment syndrome, 2× aneurysm, 1× haemolysis, 1× intracerebral haemorrhage, 1× diffuse bleeding with shock on cannulation
Liebold and colleagues [30]	2002	AV	21	7× cannula thrombosis, 1× membrane clotting, 3× lower limb ischaemia, 5× membrane plasma leakage
Guinard and colleagues [22]	1997	VV	NR	NR
Morris and colleagues [18]	1994	VV	NR	21× non-CNS haemorrhage (7 requiring discontinuation of ECCO_2_R), 4× circuit clotting
Brunet and colleagues [23]	1994	VV	18.2	1× alveolar haemorrhage, 1× diffuse bleeding
Bindslev and colleagues [31]	1991	VV	NR	1× allergic reaction
Gattinoni and colleagues [24]	1986	VV	NR	3× intra-pulmonary bleeding

AV, arteriovenous; CNS, central nervous system; CRRT, continuous renal replacement therapy; ECCO_2_R, extracorporeal carbon dioxide removal; NR, not reported; VV, venovenous.

Several studies reported on transfusion requirements in those receiving ECCO_2_R. The Xtravent trialists [19] described a significant increase in the requirement for red cell transfusion in the ECCO_2_R group, between randomisation and day 10, compared with the control group (3.7 ± 2.4 versus 1.5 ± 1.3 units, *P* < 0.05). Likewise, Morris and colleagues reported a significantly higher red cell transfusion rate in those undergoing ECCO_2_R versus controls (11.1 ± 2.3 versus 3.6 ± 0.8 l/ICU stay) [18].

### Impact on sedation/analgesia

Two studies reported on sedative/analgesic requirements [19,26]. In the Xtravent study, ECCO_2_R patients had a lower cumulative dose of opioid and benzodiazepine than those in the control group [19]. Similarly in Weber-Carstens and colleagues’ 2009 study, patients required lower doses of opioid (fentanyl: 8.9 to 4.4 mg/kg/hour) and benzodiazepine (midazolam: 0.28 to 0.19 mg/kg/hour) after 4 days of ECCO_2_R treatment [26].

### Biomarkers

The role of biomarkers in predicting severity or mortality from ARDS is not straightforward. Attempts to identify a biomarker for ARDS have not thus far been successful [36,37].

Only two of the included studies [19,21] evaluated the use of ECCO_2_R and biomarkers, and neither dataset was complete. The Xtravent study measured serum levels of proinflammatory cytokines tumour necrosis factor alpha, IL-6 and IL-8 in 20 patients who underwent ECCO_2_R and in 15 controls [19]. IL-6 levels decreased in the ECCO_2_R group in the first 24 hours. Tumour necrosis factor alpha and IL-8 were unchanged. The study by Terragni and colleagues included assessment of IL-6, IL-8, IL-1b, and IL-1ra in 10 patients with higher plateau pressure of 28 to 30, and in 15 of 22 patients with lower plateau pressures [21]. In patients with higher plateau pressures, the use of ECCO_2_R to facilitate a reduction in pressure was associated with a significant reduction in pulmonary inflammatory mediators. This association suggests that inflammatory cytokines may be a useful surrogate outcome in phase 2 studies, although it is unclear whether a change in inflammatory cytokines translates into improved clinical outcomes.

## Discussion

This comprehensive systematic review examined two RCTs and 12 observational studies that treated 495 patients with acute respiratory failure with ECCO_2_R and found a paucity of high-quality clinical trials evaluating its use. Differing modalities of ECCO_2_R were compared, with arteriovenous ECCO_2_R being used in seven studies and venovenous ECCO_2_R in seven studies.

Our findings indicate that robust data supporting the use of these devices are lacking. Both RCTs were terminated early for futility and therefore it was not possible to determine an effect on mortality. Although no mortality benefit was shown, a *post hoc* analysis of the most recent RCT [19] indicated that a subset of patients with moderately severe ARDS demonstrated a trend towards more VFDs at 60 days and a shorter ICU length of stay. This *post hoc* analysis aids in determining optimal indications for use and design of future trials.

Patients with more severe respiratory failure (PaO_2_/FiO_2_ < 150) may be an appropriate population of patients to recruit for subsequent RCTs because ECCO_2_R may have a better risk–benefit profile in more severely ill patients, who are at a high risk of dying. Data from the UK Intensive Care National Audit and Research Centre (ICNARC), which collects information from 95% of ICUs in the United Kingdom, found that more than 18,500 of approximately 130,000 mechanically ventilated patients in 2012 had PaO_2_/FiO_2_ < 150. This cohort had a 40% ICU mortality and almost 50% hospital mortality. These results provide an estimation of the population who might benefit from ECCO_2_R if proven to be effective in this cohort.

Complication rates varied across the papers but the pattern suggested that increasing familiarity led to lower rates of complications. Comparing the Zimmermann and colleagues [20] and Bein and colleagues [28] nonrandomised papers from the same single centre in Regensburg, Germany, complication rates decreased from 24.4% in the earlier period to 11.9% in the prospective study. This highlights the importance of training in the introduction of the technology, and perhaps limiting its use to sites with expertise. Earlier studies demonstrated a high rate of haemorrhagic complications due to the need for systemic anticoagulation, particularly in older venovenous circuits. Newer circuits have heparin-bonded surfaces, which obviates this need. With this novel technology, there has been a decrease in bleeding rates, as demonstrated by Knoch and colleagues in 1992 [38], although bleeding remains a risk.

The increasing red cell requirement, reported not only in the Morris and colleagues study [18] but also in the more recent Xtravent study [19], represents a meaningful concern regarding ECCO_2_R use. There is evidence that red cell transfusion is associated with increased development of ARDS [39], and increasing mortality for critically ill patients [40]. Future study of these devices should assess need for red cell transfusion carefully.

Both the Xtravent study [19] and the Weber-Carstens and colleagues study [26] showed decreased need for sedation and analgesia in patients with application of ECCO_2_R; lower sedation levels have previously been associated with shorter ventilation time, shorter ICU and hospital stays, and lower mortality rates [41,42].

Data are lacking on the cost-effectiveness of this therapy. Morris and colleagues’ study estimated an increase in hospital costs of approximately 20% per day for ECCO_2_R versus control patients [18]; this was probably an underestimate because increased technical staffing costs were not included. Gattinoni and colleagues’ study demonstrated that ECCO_2_R in the 1980s was associated with a doubling of cost per day of ICU therapy [24]. Bindslev and colleagues’ 1991 study estimated that the daily cost of a patient on extracorporeal assistance is approximately three times greater than that for a standard ICU bed [31]. None of the other studies discussed here included a formal economic analysis. The potential benefits of this therapy are not yet proven, and certainly concerns still exist about its efficacy, side effects and costs. Further clinical trials should also include a health economic analysis to determine the cost-effectiveness of ECCO_2_R.

Carbon dioxide removal is quantitatively possible using strategies of ECCO_2_R, as reported in almost all of the studies. The Xtravent RCT and recent nonrandomised studies demonstrate that ventilation with very low tidal volume ventilation was feasible and safe with ECCO_2_R. While in some models respiratory acidosis has been shown to decrease pulmonary inflammatory cytokines [43], this may be attributable to an improvement in shear stress, and the role of hypercapnic acidosis in this setting is incompletely understood. Robust studies have demonstrated reductions in inflammatory cytokines [21,44] and improved outcomes [3] as a consequence of lower tidal volume ventilation. In addition, in the clinical situation, where respiratory and metabolic acidosis may co-exist, buffering or control of acidosis may be required in patients intolerant of acidaemia; for example, traumatic brain injury. This is an important point in considering the design of future clinical trials of ECCO_2_R where the primary objective should be limitation of injurious ventilation rather than reversal of respiratory acidosis.

The optimal timing for use of ECCO_2_R remains unclear, although retrospective analysis has shown that a shorter period of mechanical ventilation (3 versus 5 days) before application of the device is associated with an improved mortality rate [20]. One group used ECCO_2_R as a rescue strategy following prolonged ventilation (9.9 ± 6.2 days) [22] and mortality rates were particularly high in this group (75%).

The strength of this review is that we adhered closely to our protocol, which outlined our procedures for minimising bias in the review: these included independent screening for study inclusion, data extraction and assessment of quality by two authors. With the assistance of an experienced librarian, we conducted a thorough search strategy and believe we have identified all relevant studies.

Although the recent RCT has added useful information on current ECCO_2_R use as a well-designed multicentre study with a low risk of bias [19], there is still a paucity of high-quality evidence in this area, and a need for adequately powered clinical studies. This is an area where rapid technological advances have been made in the last 10 years, representing major changes in the component sophistication and efficacy of ECCO_2_R. This confounds attempts to compare devices when a systematic review is attempted. Differences in vascular access, pumped versus nonpumped systems, cardiovascular status of the patient, need for full anti-coagulation, and more efficient modern extraction abilities all complicate analysis.

Our work has some limitations, including those common to all systematic reviews. We are reliant on the available evidence, and over one-half of included studies were case series, which are graded as 3 (that is, low-quality evidence) [45] (Table 2). Only two RCTs on the use of ECCO_2_R (graded as level 1+) were conducted, both of which stopped early and had a time interval of 19 years between them and associated differences in practice. As such, they offer limited information to inform practice. A formal meta-analysis of the data was not possible because there were only two RCTs with significant heterogeneity within those studies.

The UK National Institute of Clinical Evidence guidelines on ECCO_2_R state that ‘evidence on its efficacy is limited in quantity and quality’ [46]. The 2010 Canadian Health Technology Assessment found that arteriovenous ECCO_2_R is efficacious regarding carbon dioxide removal and can therefore facilitate lung protective ventilator strategies, but like our review it found no evidence of improved long-term survival [34].

We found two ongoing studies. First is a clinical trial that is currently recruiting in Turin, with the aim of comparing ultra-low tidal volume ventilation (4 ml/kg predicted body weight) with low tidal volume ventilation (6 ml/kg predicted body weight), using ECCO_2_R to facilitate this [47]. This is a randomised nonblinded study, with the aim to recruit 230 patients over 12 to 18 months. The other ongoing study is recruiting to assess ECCO_2_R combined with early renal replacement therapy [48]. The Xtravent study was conducted in 10 centres over 40 months and yet only 79 patients were enrolled [19], underlining the difficulties of enrolment in this patient population.

Our review indicates a state of clinical equipoise on the benefits of ECCO_2_R in acute respiratory failure. There is a trend towards improved outcomes as indicated by more VFDs with the application of modern ECCO_2_R in patients with more severe disease; however, definitive data are as yet lacking. Questions regarding the optimal device, timing and patient selection will be best answered by further rigorous and well-designed RCTs.

## Conclusions

ECCO_2_R is an area of rapidly evolving technology and increasing rates of utilisation. As physicians, nurses and technical staff gain familiarity with this technology, its use is likely to increase. As a treatment modality for respiratory failure and ARDS, ECCO_2_R is efficacious as a treatment for hypercapnia, facilitating ultra-low tidal volume ventilation. There is some indication that patients with moderately severe disease in whom ECCO_2_R is employed at an early stage may benefit. However, evidence for a reduction in mortality and other important clinical outcomes is still lacking.

## Abbreviations

ARDS: Acute respiratory distress syndrome; ECCO2R: Extracorporeal carbon dioxide removal; FiO2: Fraction of inspired oxygen; ICNARC: Intensive Care National Audit and Research Centre; IL: Interleukin; LILACS: Literatura Latino Americana em Ciências da Saúde; PaO2: Partial pressure of arterial oxygen; RCT: Randomised controlled trial; VFD: Ventilator-free day.

## Competing interests

The authors declare that they have no competing interests.

## Authors’ contributions

JJM, DFM, MF and BB conceived the review. All authors made a substantial contribution to the protocol development. MF and JM extracted the data and drafted the manuscript. All authors were involved in analysis and interpretation of data, critically revised the manuscript and approved the final manuscript.

## Supplementary Material

Additional file 1 The full MEDLINE search strategy. Click here for file

Additional file 2 The pre-piloted data extraction form. Click here for file

Additional file 3 A table presenting excluded articles [49-54]. Click here for file
