# Supplementary material for: Extracorporeal carbon dioxide removal for patients with acute respiratory failure secondary to the acute respiratory distress syndrome: a systematic review
Source: Crit Care. 2014 May 15;18(3):222. doi: 10.1186/cc13875 (PMC4056779; doi:10.1186/cc13875)
Supplement: Additional file 1 — The full MEDLINE search strategy. [file cc13875-S1.docx]

**Search Strategy (**as run Ovid MEDLINE(R) and Ovid Embase(R) (1976 to 30 January 2014))

1: "interventional lung assist*".mp. [mp=title, abstract, original title, name of substance word, subject heading word, keyword heading word, protocol supplementary concept, rare disease supplementary concept, unique identifier] (59)

2: extracorporeal adj (CO2 or "carbon dioxide") adj removal).mp. [mp=title, abstract, original title, name of substance word, subject heading word, keyword heading word, protocol supplementary concept, rare disease supplementary concept, unique identifier]

3: ILA*.mp. [mp=title, abstract, original title, name of substance word, subject heading word, keyword heading word, protocol supplementary concept, rare disease supplementary concept, unique identifier]

4: novalung*.mp. [mp=title, abstract, original title, name of substance word, subject heading word, keyword heading word, protocol supplementary concept, rare disease supplementary concept, unique identifier]

5: PECLA*.mp. [mp=title, abstract, original title, name of substance word, subject heading word, keyword heading word, protocol supplementary concept, rare disease supplementary concept, unique identifier]

6: "percutaneous extracorporeal lung assist*".mp. [mp=title, abstract, original title, name of substance word, subject heading word, keyword heading word, protocol supplementary concept, rare disease supplementary concept, unique identifier]

7: "partial extracorporeal support*".mp. [mp=title, abstract, original title, name of substance word, subject heading word, keyword heading word, protocol supplementary concept, rare disease supplementary concept, unique identifier] (0)

8: (("carbon dioxide" or CO2) adj dialysis*).mp. [mp=title, abstract, original title, name of substance word, subject heading word, keyword heading word, protocol supplementary concept, rare disease supplementary concept, unique identifier] (5)

9: ECCO2R*.mp. [mp=title, abstract, original title, name of substance word, subject heading word, keyword heading word, protocol supplementary concept, rare disease supplementary concept, unique identifier] (43)

10: "low flow ECCO2R*".mp. [mp=title, abstract, original title, name of substance word, subject heading word, keyword heading word, protocol supplementary concept, rare disease supplementary concept, unique identifier] (1)

11: 1 or 2 or 3 or 4 or 5 or 6 or 7 or 8 or 9 or 10 or 11

12: exp Intensive Care Units

13: exp Intensive Care

14: exp Critical Care

15: (ICU* or ITU*).mp. [mp=title, abstract, original title, name of substance word, subject heading word, keyword heading word, protocol supplementary concept, rare disease supplementary concept, unique identifier]

16: 12 or 13 or 14 or 15

17: Respiratory Distress Syndrome, Acute

18: 11 and 16 and 17

19: limit 18 to humans
