# Supplementary material for: Extracorporeal carbon dioxide removal for patients with acute respiratory failure secondary to the acute respiratory distress syndrome: a systematic review
Source: Crit Care. 2014 May 15;18(3):222. doi: 10.1186/cc13875 (PMC4056779; doi:10.1186/cc13875)
Supplement: Additional file 2 — The pre-piloted data extraction form. [file cc13875-S2.docx]

ECCO_2_R Systematic Review

**DATA EXTRACTION + RANK OF BIAS ASSESSMENT FORM**

| Title | First author | Journal | Year |
| --- | --- | --- | --- |
|  |  |  |  |

**Study eligibility**

| RCT/Quasi/CCT | Adult patients, RF | ECCO2R removal type | Mortality outcomes |
| --- | --- | --- | --- |
|  |  |  |  |

| Do not proceed if any of the above answers are ‘No’. If the study meets the inclusion criteria, but is subsequently excluded, please detail the reasons for exclusion. |
| --- |
|  |

Participants and trial characteristics

| **Participant characteristics** | |
| --- | --- |
|  | n = |
| Age (mean, median, range, etc) |  |
| Sex of participants (numbers / %, etc) |  |
| Disease status / type, etc (if applicable) |  |
| Other |  |

Trial characteristics

| Single centre / multicentre |  |
| --- | --- |
| Country / Countries |  |
| How was participant eligibility defined? |  |
| How many people were randomised? |  |
| Number of participants in each intervention group | Intervention:  Control: |
| Number of participants who received intended treatment | Intervention:  Control: |
| Number of participants who were analysed | Intervention:  Control: |
| Treatment Group – ECC0_2_R Mode/Model |  |
| Control Group Intervention |  |
| Duration of treatment (State weeks / months, etc, if cross-over trial give length of time in each arm) |  |
| Median (range) length of follow-up reported in this paper (state weeks, months or years or if not stated) |  |
| Time-points when measurements were taken during the study |  |
| Time-points reported in the study |  |
| Trial design (e.g. parallel / cross-over*) |  |
|  |  |

The Cochrane Collaboration’s tool for assessing risk of bias

| **Domain** | **Description** | **Review authors’ judgment** |
| --- | --- | --- |
| **Sequence generation** |  | Was the allocation sequence adequately generated?  YES / NO / UNCLEAR |
| **Allocation concealment** |  | Was allocation adequately concealed?  YES / NO / UNCLEAR |
| **Incomplete outcome data** *Assessments should be made for each main outcome (or class of outcomes)* |  | Were incomplete outcome data adequately addressed?  YES / NO / UNCLEAR |
| **Selective outcome reporting** |  | Are reports of the study free of suggestion of selective outcome reporting?  YES / NO / UNCLEAR |
| **Other sources of bias** |  | Was the study apparently free of other problems that could put it at a high risk of bias?  YES / NO / UNCLEAR |

**Data extraction**

| **Outcomes relevant to your review**  Copy and paste from ‘Types of outcome measures’ | |
| --- | --- |
|  | Reported in paper |
| Mortality | Y/N |
| Ventilator free days | Y/N |
| Duration of ICU stay | Y/N |
| CO2 removal (quantified) | Y/N |
| Organ-failure free days | Y/N |
| Complications | Y/N |

| **For Continuous data** | | | | | | |
| --- | --- | --- | --- | --- | --- | --- |
| Outcomes | Unit of measurement | Intervention group | | Control group | | Details if outcome only described in text  P value, 95% CI |
|  |  | n | Mean (SD) | n | Mean (SD) |  |
| Charactersistics of patients who survived |  |  |  |  |  |  |
| CO2 Removal | Mm Hg |  |  |  |  |  |
| MAP | mm Hg |  |  |  |  |  |
| Norad | microg/kg/min |  |  |  |  |  |
| Peak ins pr | cm H2O |  |  |  |  |  |
| Complications |  |  |  |  |  |  |
| MV | L/min |  |  |  |  |  |

| **For Dichotomous data** | | |
| --- | --- | --- |
| Outcomes | Intervention group (n)  n = number of participants, not number of events | Control group (n)  n = number of participants, not number of events |
| Mortality |  |  |
|  |  |  |
|  |  |  |
|  |  |  |
|  |  |  |
|  |  |  |

| **Other information which you feel is relevant to the results**  Indicate if: any data were obtained from the primary author; if results were estimated from graphs etc; or calculated by you using a formula (this should be stated and the formula given). In general if results not reported in paper(s) are obtained this should be made clear here to be cited in review. |
| --- |
|  |

References to other trials

| Did this report include any references to published reports of potentially eligible trials not already identified for this review? | | | |
| --- | --- | --- | --- |
| Title | First author | Journal / Conference | Year of publication |
|  |  |  |  |
|  |  |  |  |
|  |  |  |  |
|  |  |  |  |
| Did this report include any references to unpublished data from potentially eligible trials not already identified for this review? If yes, give list contact name and details | | | |
|  | | | |
