# Supplementary material for: Extracorporeal carbon dioxide removal for patients with acute respiratory failure secondary to the acute respiratory distress syndrome: a systematic review
Source: Crit Care. 2014 May 15;18(3):222. doi: 10.1186/cc13875 (PMC4056779; doi:10.1186/cc13875)
Supplement: Additional file 3 — A table presenting excluded articles [49-54]. [file cc13875-S3.docx]

**Additional file 3 Excluded papers**

| Author | **Year** | **Journal** | **Title** | **Reason for exclusion** |
| --- | --- | --- | --- | --- |
| Atalan et al ^33^  Mattei et al ^32^  Muller et al ^51^ | 2013    2011  2009 | Critical Care  European Journal of Anaesthesiology  European Respiratory Journal | Combined use of pumpless extracorporeal lung assist system and continuous renal replacement therapy with citrate  anticoagulation in polytrauma patients  Extracorporeal CO_2_ removal in ICU  Extracorporeal pumpless interventional lung assist in clinical practice: determinants of efficacy | Limited data available  Limited data available  Repetition of dataset - previously published |
| Bein T et al ^50^ | 2004 | Anaesthesist | Pumpless extracorporeal lung assist using arterio-venous shunt in severe ARDS. Experience with 30 cases | Repetition of dataset - subsequently published |
| Belghith et al ^49^ | 1993 | La Revue du Praticien | Low frequency positive-pressure ventilation and extracorporeal CO2 removal. Treatment of acute respiratory distress syndrome in adults. | Review article (no abstract available) |
| Sittig DF et al ^52^ | 1989 | Computer Methods & Programs in Biomedicine | Computerized management of patient care in a complex, controlled clinical trial in the intensive care unit. | Study Protocol – no outcome data |
| Marcolin et al ^53^ | 1986 | ASAIO transactions / American Society for Artificial Internal Organs | Ventilatory impact of partial extracorporeal CO2 removal (PECOR) in ARF patients | Review article (no abstract available) |
| Hickling K.G. ^54^ | 1986 | Anaesthesia and Intensive Care | Extracorporeal CO2 Removal in severe adult respiratory distress syndrome. | Review article |

References

49. Belghith M, Brunet F: [**Low frequency positive-pressure ventilation and extracorporeal CO_2_ removal. Treatment of acute respiratory distress syndrome in adults**] **Ventilation apneique et epuration extracorporelle de CO2. Traitement du syndrome de detresse respiratoire aigue de l'adulte.** *Rev Prat* 1993, **43:**2089-2092.

50. Bein T, Prasser C, Philipp A, Müller T, Weber F, Schlitt HJ, Schmid FX, Taeger K, Birnbaum D: **Pumpless extracorporeal lung assist using arterio-venous shunt in severe ARDS. Experience with 30 cases.** *Anaesthesist* 2004, **53:**813-819.

51. Müller T, Lubnow M, Philipp A, Bein T, Jeron A, Luchner A, Rupprecht L, Reng M, Langgartner J, Wrede CE, Zimmermann M, Birnbaum D, Schmid C, Riegger GA, Pfeifer M: **Extracorporeal pumpless interventional lung assist in clinical practice: determinants of efficacy.** *Eur Respir J* 2009, **33:**551-558.

52. Sittig DF, Gardner RM, Pace NL, Morris AH, Beck E: **Computerized management of patient care in a complex, controlled clinical trial in the intensive care unit.** *Comput Methods Programs Biomed* 1989, **30:**77-84.

53. Marcolin R, Mascheroni D, Pesenti A, Bombino M, Gattinoni L: **Ventilatory impact of partial extracorporeal CO2 removal (PECOR) in ARF patients.** *ASAIO Trans* 1986, **32:**508-510.

54. Hickling KG: **Extracorporeal CO_2_ removal in severe adult respiratory distress syndrome.** *Anaesthesia Intensive Care* 1986, **14:**46-53.
